# Assessment of a Medical Student–Run Multidisciplinary Oncology Shadowing Program

**DOI:** 10.1007/s13187-024-02522-w

**Published:** 2024-10-16

**Authors:** Marco Santos Teles, Shrey B. Shah, Malcolm D. Mattes

**Affiliations:** 1 Rutgers New Jersey Medical School, Newark, NJ USA; 2 Department of Radiation Oncology, Rutgers Cancer Institute of New Jersey, 195 Little Albany Street, New Brunswick, NJ 08901 USA

**Keywords:** Undergraduate medical education, Clinical oncology, Radiation oncology, Medical student

## Abstract

Clinical exposures during the first two years of medical school (MS1-2) provide students an opportunity for early networking, mentorship and career exploration. The feasibility and perceived value of an extracurricular, student-run multidisciplinary oncology shadowing program was evaluated in this study. At a single institution, an oncology interest group collaborated with oncologists to create a shadowing program for MS1-2 students. Three radiation oncology (RO), two medical oncology (MO), and four surgical oncology (SO) faculty participated. A quarterly sign-up form was emailed to all MS1-2 students with available shadowing sessions. Electronic surveys were sent to students (after shadowing) and faculty (after 18 months of program operation) with multiple choice and 5-point Likert-type (1 = low, 5 = high) questions about their experiences. A total of 57 of the 97 participating students, and all 9 of the 9 participating faculty, responded to surveys (response rates 59% and 100%, respectively). Thirty students (53%) shadowed in RO, 23 (40%) in SO, and 4 (7%) in MO. Most students were very satisfied with their interactions with faculty (mean Likert-type rating of 4.14 ± standard deviation 0.85) and their overall experience compared to prior shadowing experiences (3.84 ± 0.85). Fifty-four students (96%) would recommend the experience to a classmate. Four faculty (44%) reported much more shadowing than before. Seven faculty (78%) felt the experience was best when residents or fellows were also present. All physicians planned to continue participating. This study demonstrated that a student-run, oncology shadowing program was feasible to implement, and viewed favorably by faculty and student participants.

**Supplementary Information:**

The online version contains supplementary material available at 10.1007/s13187-024-02522-w.

## Introduction

Opportunities for medical students to have clinical exposures during the first and second (preclinical) years of medical school is important for a variety of reasons. These experiences can help increase students’ comfort level with patient care prior to their clinical clerkships [1, 2]. Students can also explore a variety of specialties that they may not have exposure to during their clinical clerkships, to guide their career decisions. Early networking and mentorship can also help position students to be more competitive residency applicants [3].While medical schools may offer a longitudinal preceptorship experience during the preclinical curriculum, such experiences may limit students to a single mentor or be more primary care oriented [4–6]. In theory, students may create shadowing opportunities in any specialty of their choosing simply by contacting faculty directly; however, many students may be intimidated by this, or not know where to begin.

Development of formal extracurricular shadowing programs may have several advantages, including connecting students with faculty in fields of medicine that interest them, and streamlining the coordination of administrative processes Such a program may be particularly valuable for giving students early exposure to clinical oncology, given that aspects of clinical cancer care are often fragmented or under-emphasized in US medical school curricula [4–8], and because most oncology exposure during the clinical clerkships involves the care of inpatients, whereas most of oncologic care takes place on an outpatient basis [8]. Student-run initiatives, such as oncology interest groups, are known to effectively provide extracurricular learning and exposure in oncology, but more commonly through didactics than direct patient care [2]. In this pilot study, we aim to assess the feasibility and overall impact of implementing a student-run multidisciplinary oncology shadowing program for first- and second-year medical students.

## Methods

The shadowing program was created with two primary educational objectives: (1) to provide pre-clinical medical students with exposure to oncological subspecialties and (2) to increase student interest in those subspecialties. In May 2022, a student-run oncology interest group at a single allopathic US medical school collaborated with oncologists at its affiliated cancer center to create an oncology shadowing program for first- and second-year medical students. All 6 medical oncologists, 3 radiation oncologists, and 10 surgical oncologists were invited to participate in the program to host medical student shadowing at their clinic.

Ultimately 2 medical oncologists, 3 radiation oncologists, and 4 surgical oncologists (in the fields of breast surgery, colorectal surgery, and urology) agreed to participate. The medical oncologists offered 5 shadowing sessions per month (1 oncologist offered weekly sessions and the other every 3 weeks), the radiation oncologists 12 per month (each weekly), and the surgical oncologists 20 per month (3 weekly and 1 twice a week). A quarterly sign-up form was then sent to all medical students via email with the available shadowing sessions with these oncology subspecialists. Each faculty member was asked to provide instructions on expectations, contact information, location of the clinic, and any pre-shadowing educational materials recommended, for the students to review in advance. The surgical and medical oncologists did not provide any pre-shadowing materials to review, whereas students shadowing in radiation oncology received a link to a 30-min introductory video about the specialty from the American Society for Radiation Oncology (ASTRO).

Each shadowing session lasted approximately 3–4 h. During each session, students spent the day with an attending oncologist, observing a variety of clinical activities. A list of clinical activities that students observed during the shadowing sessions is shown in Table 1. At minimum, students observed new and follow-up patient visits, treatment planning and informed consent discussions, and patient education/counseling. In some cases, students also observed clinical activities specific to each subspecialty that happened to occur that day (e.g., surgical procedures in clinic, brachytherapy, radiation treatment planning). When possible, the oncologist spent time discussing important teaching points for the patient encounters, which involved introductory concepts of clinical oncology (e.g., staging, diagnosis, treatment modalities and strategies). Residents and fellows were sometimes also involved in mentoring students if they were working with a given faculty member on a shadowing day. Students were paired with them for initial patient history intake, followed by discussion of the case with the attending oncologist. Students participated in the program from August 2021 to December 2023.

**Table 1:** Overview of clinical exposure and instruction of shadowing experiences

Oncological subspecialty	Educational discussions	Clinical activities observed	
		*Routine activities*	Variable activities*
**Medical Oncology**	• Introduction to concepts of clinical oncology (e.g., cancer biology, diagnosis, staging, treatment modalities) • Fundamental concepts of staging and prognosis (clinical vs. pathological staging, localized vs. metastatic disease) • Basic understanding of treatment intent (curative vs. palliative) and strategies (e.g., adjuvant, neoadjuvant, maintenance therapy)	• New and follow-up patient visits, including: - history taking - physical exam - symptom control - diagnostic test review • Treatment planning and informed consent discussions • Patient education and counseling	• Inpatient consultations • End-of-life/goals of care discussions • Bone marrow aspirations
**Radiation Oncology**			• On-treatment and simulation visits • Radiation treatment planning (contouring) • Brachytherapy
**Surgical Oncology**			• In-office procedures (e.g., fine-needle aspirations, biopsies, endoscopies) • Post-surgical wound care (e.g., suture and drain removal, dressing changes)

^*^These activities were only a part of the student’s experience if they were in the physician’s schedule when the shadowing session took place

At the end of each session, students were invited to complete an electronic survey built using Research Electronic Data Capture (REDCap). Students were asked several questions in a 5-point Likert-type scale about their satisfaction with the program, level of interest/exposure in the subspecialty before and after the experience, and perceived increase in clinical knowledge. The final section of the survey collected basic demographic information from students. Supplementary Fig. 1 shows all the questions in this survey. The Likert-type scale was defined as 1 = not at all to 5 = extremely for questions assessing specialty interest, satisfaction with the program, perceived knowledge increase and enhancement of pre-clinical curriculum (e.g., not at all interested to extremely interested). For questions comparing the program to other experiences, the Likert-type scale was defined as 1 = much worse, 3 = neutral, and 5 = much better. The survey was anonymous, and participation was voluntary. A $10 e-gift card was provided as compensation to students after completing the survey. Non-responders received up to 3 reminder emails.

After the program had been operating for 18 months, all participating physicians were also asked to complete an electronic survey about their experience via REDCap. Physicians were asked to share their opinion about multiple aspects of the program, related to the work involved in hosting students, feasibility, teaching preferences, satisfaction with the program, and ways to improve the experience. The questions used a 5-point Likert-type scale, multiple choice, or free text format. Supplementary Fig. 2 shows all the questions in this survey. The Likert-type scale was defined as 1 = not at all to 5 = extremely for questions assessing aspects of the program. The survey was sent between December 2023 and January 2024. Institutional Review Board (IRB) approval was obtained prior to data collection. For both surveys, descriptive statistics were used to report participants’ Likert-type responses and demographics. Students’ mean Likert-type ratings for each aspect of the program are reported. Non-parametric tests were used to compare differences between the Likert-type responses of participants, to account for the small sample size, non-normal distribution, and ordinal nature of the data. The Wilcoxon signed-rank test was used to compare differences in perceived interest level before and after the shadowing experience. The Friedman test was used to compare differences in physicians’ teaching preferences.

## Results

A total of 97 students participated in the shadowing program, and 57 students responded to the questionnaire (response rate 58.8%). Their demographics are shown in Table 2. The majority of students were female, in their first year of medical school, shadowed in radiation oncology, and had a resident or fellow present with the faculty during the shadowing. Students’ prior exposures to oncology before the shadowing experience are shown in Table 3. Most students had no prior exposure to any of the three oncological subspecialties. A total of 48 students (84.2%) prepared for the experience by reading provided material or reviewing coursework pertaining to the specialty, and 41 students (80.4%) reported no problems in signing-up for the shadowing experience. Students spent a median of 3.0 h (interquartile range: 3.0–4.0) in each shadowing session.

**Table 2:** Student demographics and basic information about shadowing experience

		*n* (%)
Gender	Female	34 (59.6%)
	Male	22 (38.6%)
	Prefer not to answer	1 (1.8%)
Year of medical school	First year	44 (77.2%)
	Second year	13 (22.8%)
Subspecialty shadowed	Medical Oncology	4 (7.0%)
	Radiation Oncology	30 (52.6%)
	Surgical Oncology	23 (40.4%)
Resident or fellow present?	Yes	45 (78.9%)
	No	12 (21.1%)

**Table 3:** Students’ prior exposures to oncology before shadowing experience

Oncological subspecialty		*n* (%)
Oncology	Prior shadowing in the specialty	6 (11.3%)
	Friends or family that work in the specialty	1 (1.8%)
	Prior research experience in the specialty	9 (17.0%)
	Attended a presentation from faculty in the specialty	9 (17.0%)
	No previous exposure	28 (52.8%)
Radiation Oncology	Prior shadowing in the specialty	1 (1.8%)
	Friends or family that work in the specialty	4 (7.1%)
	Prior research experience in the specialty	1 (1.8%)
	Attended a presentation from faculty in the specialty	10 (17.9%)
	No previous exposure	40 (71.4%)
Surgical Oncology	Prior shadowing in the specialty	4 (7.1%)
	Friends or family that work in the specialty	1 (1.8%)
	Prior research experience in the specialty	4 (7.1%)
	Attended a presentation from faculty in the specialty	11 (19.6%)
	No previous exposure	36 (64.3%)

Students’ ratings of multiple aspects of the overall impact of the program are shown in Table 4. Most students were satisfied with the quality of their interactions with both faculty (mean Likert-type rating of 4.14 ± 0.85) and patients (3.84 ± 0.98) during the experience. Compared to other previous shadowing experiences, most students rated this program more favorably in terms of the overall experience (3.84 ± 0.85), as well as educational content (3.50 ± 0.74), exposure to the field (3.68 ± 0.82), organization (3.56 ± 0.93), and satisfaction (3.68 ± 0.87). Most students wanted to seek out further oncology shadowing with the program (3.75 ± 1.08), and 96.4% (N = 54/56) of students would recommend this shadowing experience to a classmate. Students perceived a moderate increase in clinical knowledge (3.30 ± 1.02) and enhancement of learning from their preclinical curriculum (3.04 ± 1.00) from the experience. Students had an overall moderate interest in all three oncological subspecialties prior to the experience, but those who shadowed in radiation oncology reported a significantly higher level of interest in the specialty after the experience (2.80 ± 0.887 vs. 3.33 ± 1.028, *p* = 0.015).

**Table 4:** Students’ Mean (standard deviation) and Median (interquartile range) Likert-type rating of aspects of the shadowing experience

		n	Mean (SD)	Median (IQR)	*p-*value
What was your level of interest in medical oncology?*	Before shadowing	4	3.75 (0.957)	3.50 (3.00–4.75)	0.317
	After shadowing	4	4.00 (0.816)	4.00 (3.25–4.75)	
What was your level of interest in radiation oncology? *	Before shadowing	30	2.80 (0.887)	3.00 (2.00–3.00)	0.015
	After shadowing	30	3.33 (1.028)	3.00 (2.00–4.00)	
What was your level of interest in surgical oncology?*	Before shadowing	23	3.52 (1.082)	4.00 (3.00–4.00)	0.592
	After shadowing	23	3.65 (1.152)	4.00 (3.00–5.00)	
How satisfied did you feel with the quality of your interactions?	With patients	57	3.84 (0.98)	4.00 (3.00–5.00)	
	With faculty	57	4.14 (0.85)	4.00 (4.00–5.00)	
Compared with other shadowing experiences, please rate the oncology shadowing program in terms of:	Educational content	50	3.50 (0.74)	3.00 (3.00–4.00)	
	Exposure to the field	50	3.68 (0.82)	3.50 (3.00–4.00)	
	Organization	50	3.56 (0.93)	3.00 (3.00–4.00)	
	Satisfaction	50	3.68 (0.87)	3.50 (3.00–4.00)	
	Overall value of the experience	50	3.84 (0.85)	4.00 (3.00–5.00)	
How has this experience increased your clinical knowledge?		57	3.30 (1.02)	3.00 (2.00–4.00)	
How has this experience enhanced your preclinical curriculum?		57	3.04 (1.00)	3.00 (2.50–4.00)	
How likely are you to seek out further shadowing?		56	3.75 (1.08)	4.00 (3.00–5.00)	

*SD*, Standard Deviation; *IQR*, interquartile range

^*^Mean Likert reported only for students who shadowed in those oncological subspecialties

A total of 9 physicians volunteered to participate in the program, and all 9 physicians responded to the mentor experience survey (response rate 100%). Physicians had been in practice for a median 7.5 years since residency or fellowship completion (IQR: 4.5–11.5). All 9 physicians noticed at least a slight increase in student shadowing since the initiation of the program, with four of them (44.4%) reporting much more students shadowing than before. Seven of the physicians (77.8%) found no difficulty in providing patient care with the students present for shadowing, but five of them (55.6%) had to limit the number of students shadowing, citing space limitation and hosting other students for clerkships as the most common reasons. Five of the physicians (55.6%) preferred to host student shadowing when residents or fellows were present in the clinic, and seven of the physicians (77.8%) thought students learn more when residents or fellows are present during shadowing. Five of the physicians (55.6%) thought the shadowing program had equal educational value for both MS1 and MS2 students, whereas 3 (33.3%) felt it was more beneficial for second year medical students. Physicians generally preferred teaching students who were further along in their training, rating residents/fellows in their specialty as most enjoyable to teach (4.89 ± 0.601), followed by MS3/4 (4.11 ± 0.928), MS1/2 (3.11 ± 1.30), and high school/college students (3.00 ± 1.42), a statistically significant difference (*p* < 0.001). All 9 physicians planned to continue hosting student shadowing for the program.

## Discussion

Shadowing experiences during the preclinical years of medical school may offer early career exploration, networking, mentorship, and clinical exposure to medical students. In this study, we have demonstrated that implementing a student-run extracurricular multidisciplinary oncology shadowing program for first and second-year medical students is feasible and well-received by faculty and student participants alike. Our preliminary assessment suggests that the program provided valuable clinical oncology experiences to students with limited prior exposure and may also enhance interest in underrecognized oncological subspecialties, such as radiation oncology. Although our sample size was relatively small in this preliminary report, our findings may also suggest ways to improve future shadowing programs and related educational initiatives.

To our knowledge, there have been no prior studies implementing a student-run, extracurricular, and multidisciplinary shadowing experience for pre-clinical medical students in oncology. However, there have been similar student-led educational initiatives. Haupt et al. reported that a 2-week pre-clerkship residency exploration program with half day clinical rotations in multiple specialties increased self-reported interest in radiation oncology [2]. Agarwal et al. described the positive impact of a student-run multidisciplinary interest group that offered oncology-related opportunities for students, including shadowing experiences [9]. There are also student-led shadowing programs for other specific specialties, which have demonstrated increased interest in that specialty [10, 11]. Other structures of early clinical experience programs in oncology have also been reported to positively impact students [12–15], though our study also considers the faculty response to their participation in the program. Our study adds to the existing literature by demonstrating the feasibility of these types of student-led shadowing programs in oncology, while also exploring physicians’ experiences and preferences when mentoring students during these educational activities.

There are several limitations to this study. Although we had a favorable response rate from the students and faculty, selection bias is inherent to any study of this nature, since those students who responded to the survey may have had a more favorable impression of their shadowing experience than those who did not. It is also likely that other medical students with less baseline interest in oncology would have responded differently, though this is not necessarily a limitation given that this extracurricular program was specifically designed for students with an interest in oncology to explore it further. More favorable responses to the survey may also have been biased by social desirability or the financial compensation offered for completion of the survey. Of note, the financial incentive was not advertised to students as an incentive to participate in the shadowing itself (only the survey completion), so we feel it was unlikely to have encouraged more students to participate in the program. The survey could have also been improved with inclusion of more robust qualitative questions to better contextualize students and faculty perceptions of the program. There were also some limitations to our structuring of the program itself. For instance, although some efforts were made to have a basic format and duration for all student shadowing experiences, inevitably there was some variability in the number and type of patient encounters and special procedures that students would have from day to day in an extracurricular program such as this. This variability in duration and format may have led to some bias in the survey findings. Our sample size was also small, particularly for medical oncology, which limits the generalizability of our findings and our ability to perform multivariate analysis. Finally, this study was conducted at a single center, and it is not known if this program would be feasible at all medical schools, given the different curricular structure and administrative processes of each school.

Expansion of this program to multiple institutions with further evaluation of its efficacy in a variety of academic environments is an important next step towards validating the preliminary findings reported herein. In doing so, it will be important to engage with key stakeholders at each institution, including the leaders of student run oncology interest groups and their faculty advisors. We would recommend standardized pre-shadowing educational materials for all students so that they can better understand the clinical encounters observed. Furthermore, faculty should be given clear expectations and/or training about the structure of the program prior to hosting students. Students could also be provided with checklists of educational goals and clinical activities to review with the faculty. Although this is an extracurricular experience, formalizing its structure is likely to lead to more standardized and effective learning for students [16, 17].

## Conclusion

This pilot study suggests the feasibility of a student-run, multidisciplinary oncology shadowing program for first and second-year medical students at a single-institution. Based on these preliminary data, we plan to refine and expand the program to a larger cohort of students and faculty across multiple institutions, to better assess the generalizability of our findings and mitigate potential biases in the survey responses.

## Supplementary Information

Below is the link to the electronic supplementary material.

Supplementary file1 (PDF 52 KB)

Supplementary file2 (PDF 39 KB)

## Data Availability

Research data are stored in an institutional repository and will be shared upon request to the corresponding author.
