# Supplementary material for: Assessment of a Medical Student–Run Multidisciplinary Oncology Shadowing Program
Source: J Cancer Educ. 2024 Oct 16;40(3):402–7. doi: 10.1007/s13187-024-02522-w (PMC12177014; doi:10.1007/s13187-024-02522-w)
Supplement: Supplementary file 1 — Supplementary file1 (PDF 52 KB) [file 13187_2024_2522_MOESM1_ESM.pdf]

Please indicate whether you agree to participate in this study	◯ Agree<br>◯ Do not agree
Which of the following specialties did you shadow?	◯ Medical Oncology<br>◯ Radiation Oncology<br>◯ Surgical Oncology
Which surgical subspecialty?	__________________________________
Approximately how many hours did you spend shadowing per session, during this experience?	__________________________________

What was your level of interest in each of the following specialties prior to this shadowing experience?

	1-Not at all interested	2-Slightly interested	3-Moderately interested	4-Very interested	5-Extremely interested
Medical Oncology	◯	◯	◯	◯	◯
Radiation Oncology	◯	◯	◯	◯	◯
Surgical Oncology	◯	◯	◯	◯	◯

What is your level of interest in each of the following specialties after this experience?

	1-Not at all interested	2- Slightly interested	3-Moderately interested	4- Very interested	5-Extremely interested
Medical Oncology	◯	◯	◯	◯	◯
Radiation Oncology	◯	◯	◯	◯	◯
Surgical Oncology	◯	◯	◯	◯	◯

How would you quantify your exposure to each specialty, prior to this experience ? (select all that apply)

	I have shadowed in this specialty before	I have friends or family that work in this specialty	I have research experience in this specialty	I have heard a presentation from a member of this specialty	I have no previous exposure in this specialty
Medical Oncology	☐	☐	☐	☐	☐
Radiation Oncology	☐	☐	☐	☐	☐
Surgical Oncology	☐	☐	☐	☐	☐

What did you do to prepare for this experience ?

(select all that apply)

- ☐ I was given background reading prior to the shadowing experience.
- ☐ I spent time reading about the specialty, outside of what was provided to read.
- ☐ I reviewed course material pertaining to the topic.
- ☐ Nothing

How satisfied did you feel with the quality of your interactions with faculty and/or providers?

- ◯ 1-Not at all satisfied
- ◯ 2-Slightly satisfied
- ◯ 3-Moderately satisfied
- ◯ 4-Very satisfied
- ◯ 5-Extremely satisfied

If you would like, please explain why:

____

How satisfied did you feel with the quality of your interactions with patients?

- ◯ 1-Not at all satisfied
- ◯ 2-Slightly satisfied
- ◯ 3-Moderately satisfied
- ◯ 4-Very satisfied
- ◯ 5-Extremely satisfied

If you would like, please explain why:

____

How has this experience increased your clinical knowledge?

- ◯ 1-Not at all increased
- ◯ 2-Slightly increased
- ◯ 3-Moderately increased
- ◯ 4-Significantly increased
- ◯ 5-Extremely increased

How has this experience enhanced your current or past covered preclinical curriculum?

- ◯ 1-Not at all enhanced
- ◯ 2-Slightly enhanced
- ◯ 3-Moderately enhanced
- ◯ 4-Significantly enhanced
- ◯ 5-Extremely enhanced

How likely are you to seek out further shadowing and/or networking with this physician?

- ◯ 1- Not at all likely
- ◯ 2- Slightly likely
- ◯ 3- Moderately likely
- ◯ 4- Very likely
- ◯ 5- Extremely likely

Compared with other shadowing experiences you had this year, please rate the oncology shadowing program in terms of :

	Much worse	Somewhat worse	About the same	Somewhat better	Much better
Educational content:	◯	◯	◯	◯	◯
Exposure to the field:	◯	◯	◯	◯	◯
Organization:	◯	◯	◯	◯	◯
Satisfaction:	◯	◯	◯	◯	◯
Overall Value of the Experience:	◯	◯	◯	◯	◯

Were there any residents present during your shadowing experience?

◯ Yes
◯ No

Did you encounter any of the following problems when signing-up for this shadowing experience ?

(Select all that apply)

☐ I could not sign-up for my preferred shift and/or specialty
☐ I was on the waitlist
☐ I did not have any problems in signing-up for this experience.
☐ Other

If other, please specify:

____

Would you recommend this experience to classmates?

◯ Yes
◯ No

Do you have any suggestions for improving this program?

____

What year of medical school are you in?

◯ First year medical student
◯ Second year medical student
◯ Third year medical student
◯ Fourth year medical student
◯ Research Year
◯ Pre-medical student
◯ Other

If other, please specify

____

Your gender:

◯ Female
◯ Male
◯ Transgender Female
◯ Transgender Male
◯ Gender variant/ Non-conforming
◯ Other/Not listed
◯ Prefer not to answer
