# Supplementary material for: Assessment of a Medical Student–Run Multidisciplinary Oncology Shadowing Program
Source: J Cancer Educ. 2024 Oct 16;40(3):402–7. doi: 10.1007/s13187-024-02522-w (PMC12177014; doi:10.1007/s13187-024-02522-w)
Supplement: Supplementary file 2 — Supplementary file2 (PDF 39 KB) [file 13187_2024_2522_MOESM2_ESM.pdf]

Mentor experience survey

Thank you for mentoring in the oncology shadowing program. We would appreciate if you could please take 5 minutes to complete this survey so that we can improve this educational experience for you and future mentors taking part in the program.

How prepared do you think students were to understand the patient cases at your clinic?

- ◯ 1- Not at all prepared
- ◯ 2-Slightly prepared
- ◯ 3-Moderately prepared
- ◯ 4-Very prepared
- ◯ 5-Extremely prepared

How has this shadowing program changed the number of pre-clinical medical students (MS1-2) who have shadowed in your clinic?

- ◯ Much fewer students than before
- ◯ A little fewer students than before
- ◯ About the same number of students as before
- ◯ A little more students than before
- ◯ Much more students than before

In what year of pre-clinical education do you think students have the most to gain from this experience?

- ◯ First-year of medical school
- ◯ Second-year of medical school
- ◯ The educational value is similar for both first and second year medical students

Please rank what type of students you most enjoy teaching.

	1-Not at all enjoyable	2-Somewhat enjoyable	3-Moderately enjoyable	4-Very enjoyable	5-Extremely enjoyable	Does not matter
High school or college students	◯	◯	◯	◯	◯	◯
1st/2nd year medical students	◯	◯	◯	◯	◯	◯
3rd/4th year medical students	◯	◯	◯	◯	◯	◯
Residents/fellows in my specialty	◯	◯	◯	◯	◯	◯

Do you think students learn more when residents or fellows are present during shadowing?

- ◯ Yes
- ◯ No

Do you think students learn more when other medical students are present during shadowing?

- ◯ Yes
- ◯ No

Do you prefer hosting medical students for shadowing when residents are present in your clinic?

- ◯ Yes
- ◯ No
- ◯ No preference
- ◯ Not Applicable

If yes, please indicate why:
(select all that apply)

- ☐ Residents are able to do some of the teaching to medical students while I focus on providing patient care.
- ☐ Residents are able to do some of the patient care while I focus on teaching medical students.
- ☐ Medical students are more comfortable asking questions to residents
- ☐ Medical students learn more when residents are present
- ☐ I tend to spend more time teaching everyone when a resident is present.
- ☐ Other

If other, please specify	__________________________
If not, please indicate why: (select all that apply)	☐ I have to provide education to both residents and medical students at the same time<br>☐ It is too many people to see patients when both residents and medical students are present<br>☐ Other
If other, please specify	__________________________
Was it any more difficult to provide patient care with the student present?	◯ 1-Not at any more difficult<br>◯ 2-Slightly more difficult<br>◯ 3-Moderately more difficult<br>◯ 4-Much more difficult<br>◯ 5-Extremely more difficult
Have you ever had to limit the number of students shadowing in your clinic through the program for any reason?	◯ Yes<br>◯ No
If yes, please explain why:	__________________________
Do you provide any didactic material for students to read/watch prior to their shadowing experience?	◯ Yes<br>◯ No
Why did you choose to volunteer for this shadowing program?	__________________________
Do you have any suggestions to improve this program?	__________________________
Do you plan to continue participating in this shadowing program?	◯ Yes<br>◯ No
What is your specialty?	__________________________
How many years ago did you graduate from your residency/fellowship training?	__________________________
